# Prediction of plant lncRNA by ensemble machine learning classifiers

**DOI:** 10.1186/s12864-018-4665-2

**Published:** 2018-05-02

**Authors:** Caitlin M. A. Simopoulos, Elizabeth A. Weretilnyk, G. Brian Golding

**Affiliations:** Department of Biology, McMaster University, 1280 Main Street West, Hamilton, Canada

**Keywords:** lncRNA, Classifier, Machine learning, Ensemble, Transcript

## Abstract

**Background:**

In plants, long non-protein coding RNAs are believed to have essential roles in development and stress responses. However, relative to advances on discerning biological roles for long non-protein coding RNAs in animal systems, this RNA class in plants is largely understudied. With comparatively few validated plant long non-coding RNAs, research on this potentially critical class of RNA is hindered by a lack of appropriate prediction tools and databases. Supervised learning models trained on data sets of mostly non-validated, non-coding transcripts have been previously used to identify this enigmatic RNA class with applications largely focused on animal systems. Our approach uses a training set comprised only of empirically validated long non-protein coding RNAs from plant, animal, and viral sources to predict and rank candidate long non-protein coding gene products for future functional validation.

**Results:**

Individual stochastic gradient boosting and random forest classifiers trained on only empirically validated long non-protein coding RNAs were constructed. In order to use the strengths of multiple classifiers, we combined multiple models into a single stacking meta-learner. This ensemble approach benefits from the diversity of several learners to effectively identify putative plant long non-coding RNAs from transcript sequence features. When the predicted genes identified by the ensemble classifier were compared to those listed in GreeNC, an established plant long non-coding RNA database, overlap for predicted genes from *Arabidopsis thaliana*, *Oryza sativa* and *Eutrema salsugineum* ranged from 51 to 83% with the highest agreement in *Eutrema salsugineum*. Most of the highest ranking predictions from *Arabidopsis thaliana* were annotated as potential natural antisense genes, pseudogenes, transposable elements, or simply computationally predicted hypothetical protein. Due to the nature of this tool, the model can be updated as new long non-protein coding transcripts are identified and functionally verified.

**Conclusions:**

This ensemble classifier is an accurate tool that can be used to rank long non-protein coding RNA predictions for use in conjunction with gene expression studies. Selection of plant transcripts with a high potential for regulatory roles as long non-protein coding RNAs will advance research in the elucidation of long non-protein coding RNA function.

**Electronic supplementary material:**

The online version of this article (10.1186/s12864-018-4665-2) contains supplementary material, which is available to authorized users.

## Background

Long non-protein coding RNAs (lncRNAs) represent a diverse and functionally important class of RNAs [1], and have been classically defined as transcripts longer than 200 nucleotides with little protein-coding potential [2]. Previously thought to be transcriptional noise, there is now evidence of their involvement in the development, disease, and stress responses of plants [3, 4]; however, these transcripts are also found throughout all kingdoms of life. LncRNA transcripts often lack sequence conservation within close relatives, and the evolution of these transcripts remains poorly understood, but there exists growing evidence of positional and structural conservation that may indicate selection on transcript function [5].

Unlike other non-coding RNAs, the mechanisms and functions of lncRNAs can range wildly – from epigenetic regulation, as exemplified by mouse *Xist* and human *XIST* [6, 7], to small RNA target mimics, as seen with *IPS1* and *ath-miR399* in *Arabidopsis thaliana* [8]. *COLDAIR*, a lncRNA associated with flowering, functions by remodeling chromatin and alters expression of the *FLC* locus [9]. A recent review by Ma et al. [10] suggests that most known lncRNAs regulate transcription, both in *cis* and *trans*, while others can affect translation, splicing, post-translational regulation or are classified as “other functional mechanisms.” Due to such a wide range of functionality, lncRNAs are typically classified by their position to protein coding genes as intergenic (also referred to as lincRNAs), natural antisense, or intronic [1, 10].

Notably, lncRNAs can not only be functional in their long RNA form, but also act as small RNA precursors and sources of small regulatory peptides [11–13] although extensive translation of lncRNAs has been disputed [14]. Adding to the complexity of these RNAs, some transcripts do not meet the arbitrary length cutoffs set by the classical definition for lncRNAs, such as *BC1* in mice (152nt) [15]. Even with recent developments in sequencing technologies, lncRNAs remain difficult to identify due to low, and condition-dependent and tissue-dependent expression levels [16]. Demonstrating minimal homology with close relatives [5], current research suggests these transcripts undergo fast and unclear evolution making functional predictions challenging. This lack of distinct rules for predicting and identifying lncRNAs is a likely contributor to the lack of validated plant lncRNAs.

Currently, many lncRNA prediction softwares that are available to researchers, such as PLEK [17], lncRScan-SVM [18], and COME [19], use machine learning methods trained on data consisting of lncRNA transcripts yet to be empirically validated. Without empirical validation, many of these predicted lncRNA transcripts could have no regulatory function and could be produced due to spurious transcription because of the low fidelity of RNApolII [20]. In addition, CPAT [21] and CPC2 [22] are popular softwares used to identify non-coding transcripts. These softwares are successful at quickly predicting the protein-coding potential of mRNA sequences, but are not specific to lncRNAs and are unsuitable for identifying those lncRNAs that may code for small peptides. Additionally, since the majority of lncRNA research is on animals, software packages for lncRNAs prediction often use only animal training datasets. While the exact functions of most plant and animal lncRNAs remain poorly understood, there are known differences in biogenesis and mechanisms of other non-coding RNAs, such as miRNAs [23]. As such, ignoring the few plant lncRNA transcripts with known function could hinder the potential of future plant lncRNA predictors.

Depending on the source, lncRNA databases can also fall victim to biases toward animal systems and non-validated transcripts as they are often model organism specific with a preference for humans, and rarely differentiate between validated and predicted lncRNA transcripts. These biases can be seen in the popular lncRNA databases, LNCipedia and NONCODE [24, 25].

Outputs from lncRNA software often result in thousands of unranked predictions leaving the researcher to choose the most likely candidates for empirical validation. In combination with an RNASeq experiment that can result in tens of thousands of transcripts, filtering through thousands of lncRNA predictions can be difficult and time consuming for a researcher. Objectively ranking predictions in combination with gene expression estimates can help researchers complete functional validation of lncRNAs more efficiently.

Recently, ensemble methods have become popular for approaching difficult biological problems typically solved by machine learning [26, 27]. Ensemble models work by combining multiple learners into a single model which helps to avoid over fitting and encourages generalization of the classifier. In addition to improved classification, ensemble methods also remove the difficulty in choosing the “best” model as all models can be used in a single classifier. Each individual classifier used in the construction of the overall ensemble model will have its own classification strengths, resulting in stronger and more accurate predictions when these classifiers are used in combination.

Here we describe a lncRNA predictor constructed using an ensemble of machine learning models developed for and tested on plant transcript sequences. We compared accuracy of this meta-learner trained on multiple machine learning models to the prediction ability of individual random forest and gradient boosting models making up the meta-learner. All models were trained on empirically validated lncRNAs to ensure only true lncRNA transcripts were used in each model’s training sets. We found the most successful method to be a stacking meta-learner constructed from eight stochastic gradient boosting models. This approach offers multiple advantages over those currently available as this machine learning approach prevents predictions from being constrained to the arbitrary classic definitions of lncRNAs, such as ignoring transcripts with high coding potential of small open reading frames (ORFs). In addition, our method numerically scores each prediction to help researchers focus their validation efforts on highly ranked lncRNA predictions. Finally, this approach uses the Diamond algorithm [28] that allows for efficient and fast sequence alignment in protein databases, an essential feature for lncRNA prediction.

## Methods

### Overview of classifiers

Multiple machine learning approaches to lncRNA prediction were compared to find the most accurate plant transcript classifier. Ensemble approaches were chosen due to the diversity of RNAs in the lncRNA category as these approaches are ideal for heterogeneous data. Ensemble models typically follow three main approaches: bagging, boosting, and stacking. Bagging (**b**ootstrap **agg**regat**ing**) relies on creating *n* models on bootstrapped training data, and averages predictions of all models for a final group prediction. This protocol is used in the random forest method. With boosting, such as in gradient boosting, one iteratively trains *n* learners, with each iteration attempting to reduce prediction error. The predictions are summed for a final classification. Finally, a stacking generalizer refers to training a new learner, for example by logistic regression, on the output of multiple learners. This is commonly referred to as meta-learner.

This study used all three approaches to ensemble methods, firstly by evaluating the lncRNA prediction accuracy of individual stochastic gradient boosting and random forest models. These individual models were then also combined into four ensemble classifiers explained further in the proceeding sections: 1. Arithmetic mean of scores, 2. Geometric mean of scores, 3. Majority vote, 4. Logistic regression meta-learner, and were evaluated similarly.

### Individual stochastic gradient boosting and random forest models

#### Data

Positive data remained constant in each training set and consisted of a total of 436 unique, validated lncRNA sequences downloaded from two separate lncRNA databases: 1. lncRNAdb v2.0 (http://lncrnadb.org) on November 25, 2016 and 2. lncRNAdisease (http://www.cuilab.cn/lncrnadisease) on February 15, 2017. These sources for lncRNA sequences include all available validated lncRNAs, but are heavily populated by animal systems and include only six plant lncRNA sequences.

Negative data for each training set consisted of sequences from four different species: *Homo sapiens*, *A. thaliana*, *Mus musculus*, and *Oryza sativa*. *H. sapiens* and *M. musculus* sequences were included in the negative data of the training set as these species are the source for the majority of validated lncRNAs. *H. sapiens* sequences were downloaded from Ensembl (http://www.ensembl.org) on December 19, 2016, *A. thaliana* from Araport v11 (https://araport-dev.tacc.utexas.edu) on December 16, 2016, *M. musculus* from Ensembl on March 28, 2017 and *O. sativa* from Ensembl on March 28, 2017. These data are made available in Additional file 2. To ensure that lncRNA, tRNAs, and rRNAs were removed from the negative training data, these types of sequences were downloaded from RNAcentral v6 (http://rnacentral.org) on March 28, 2017, using search terms available in Additional file 1 and were then removed from the dataset. Eight different training sets with different combinations of negative data from multiple species were used to construct eight different models and are described in Table 1. Sets denoted “A” and “B” remained constant throughout the training sets and were randomly chosen from the transcript sequences of each species. These training datasets were used in both random forest and gradient boosting methods, for a total of 16 preliminary models. The variety of training datasets was used to maximize model diversity, a requirement for the proceeding ensemble models.

**Table 1:** Negative training data sets in individual models, and corresponding accuracy, sensitivity, specificity and AUC values

Training dataset	Negative data	AUC		Accuracy		Specificity		Sensitivity	
		GB	RF	GB	RF	GB	RF	GB	RF
1	3000 *H. sapiens* (set A)	0.940	0.943	0.962	0.956	0.988	0.990	0.548	0.404
	1000 *M. musculus* (set A)								
	3000 *O. sativa* (set A)								
2	3000 *H. sapiens* (set A)	0.943	0.944	0.960	0.953	0.988	0.989	0.576	0.461
	3000 *O. sativa* (set A)								
3	3000 *H. sapiens* (set A)	0.961	0.962	0.973	0.970	0.990	0.992	0.693	0.592
	1000 *M. musculus* (set A)								
	3000 *A. thaliana* (set A)								
4	3000 *H. sapiens* (set A)	0.962	0.966	0.972	0.967	0.990	0.990	0.725	0.640
	3000 *A. thaliana* (set A)								
5	3000 *H. sapiens* (set B)	0.955	0.959	0.965	0.958	0.991	0.980	0.608	0.530
	3000 *A. thaliana* (set B)								
6	4500 *H. sapiens* (set A + 1500 seq)	0.961	0.967	0.979	0.979	0.995	0.995	0.633	0.571
	4500 *A. thaliana* (set A + 1500 seq)								
7	3000 *H. sapiens* (set A)	0.963	0.967	0.976	0.971	0.993	0.992	0.700	0.603
	4500 *A. thaliana* (set A + 1500 seq)								
8	2000 *H. sapiens* (2000 from set A)	0.964	0.965	0.968	0.965	0.988	0.990	0.695	0.619
	1000 *M. musculus* (set A)								
	3000 *A. thaliana* (set A)								

Training datasets of random forest (RF) and gradient boosting (GB) individual models are described. The positive training dataset, 436 validated lncRNAs, remained constant throughout all training datasets. Specificity, sensitivity, accuracy and AUC values were found using 10-fold cross validation of all training data

#### Feature extraction and selection

Eleven features were chosen for use in model construction:

1. mRNA length
2. ORF length
3. GC%
4. Fickett score
5. hexamer score
6. alignment identity in SwissProt database
7. length of alignment in SwissProt database
8. proportion of alignment length and mRNA length (alignment length:mRNA length)
9. proportion of alignment length and ORF length (alignment length:ORF)
10. presence of transposable element
11. sequence percent divergence from transposable element

Features were extracted using a combination of custom Python scripts and known software (CPAT [21] used for features 4 and 5, Diamond [28] used for features 6, 7, 8, 9, RepeatMasker [29] used for features 10 and 11).

##### CPAT model creation and application

As no publicly available plant CPAT model exists, two logit models were built using coding and non-protein coding RNA sequences from *A. thaliana* and *O. sativa*. Non-coding lncRNA, miRNA, snRNA, and snoRNA sequences from each species were downloaded from the Plant Non-coding RNA Database on September 26, 2016 (*A. thaliana*, 5062 sequences total) and July 14, 2017 (*O. sativa*, 4718 sequences total) [30]. Protein coding transcript sequences from each species were downloaded from Phytozome v11 [31] on August 3, 2016. In order to supply a balanced training set, 5938 *A. thaliana* and 5283 *O. sativa* protein coding sequences were randomly selected for a total of 11,000 *A. thaliana* transcripts and 10,000 *O. sativa* transcripts for CPAT model construction.

*A. thaliana* CPAT models were used for predictions in all species but *A. thaliana* itself, which used *O. sativa* CPAT models. Fickett and hexamer values from CPAT results were used as features in machine learning model construction.

##### Diamond alignment in SwissProt database

Diamond v0.8.34 [28] was used to quantify transcript sequence alignments to curated protein sequences in the SwissProt database [32] downloaded February 1, 2017 from http://www.uniprot.org/downloads. We ran Diamond in “more-sensitive” mode as we aligned full transcript sequences to the SwissProt database rather than RNASeq reads. Options for each Diamond run were as follows: -e 0.001, -k 5, –matrix BLOSUM62, –gapopen 11, –gapextend: 1, -f 6 qseqid pident length qframe qstart qend sstart send evalue bitscore.

##### RepeatMasker

RepeatMasker [29] was used to extract information on transcription element related features. The software was run on transcript sequences using default settings, and with -species set to Eukaryota.

#### Stochastic gradient boosting and random forest model construction and hyper-parameter selection

Once features were extracted, models were constructed using Python’s scikit-learn package [33]. Eight separate models were constructed using both gradient boosting and random forest approaches, for a total of 16 models differing in negative training data or machine learning algorithm (Table 1). All transposable element related features were removed after performing recursive feature elimination as they were found to be uninformative and reduced the accuracy of models. With the 9 remaining features, a nested 4-fold cross-validation grid search was performed for 30 trials in gradient boosting hyper-parameter selection with possible hyper-parameters:

- learning_rate: 0.02, 0.04, 0.06, 0.08, 0.1
- max_depth: 4, 6, 8, 10
- subsample: 0.2, 0.4, 0.6, 0.8, 1
- n_estimators: 100, 500, 1000

Random forest hyper-parameters remained constant through all models with the only change from default parameters being n_estimators = 5000 and min_samples_leaf = 20.

Models were evaluated by sensitivity, specificity, accuracy area under the curve (AUC) values using 10-fold cross validation and the caret R package [34].

### Ensemble learner construction

As gradient boosting and random forest models 1-8 were trained using eight different negative training sets, 3000 randomly selected *Zea mays* protein coding sequences were used as negative data in the construction and/or testing of each ensemble model for consistency through models. *Z. mays* was chosen as no training set contained sequences from this species and the genome is well annotated. *Z. mays* transcripts were downloaded from EnsemblPlants on April 27, 2017. Two separate values were used for the creation of each ensemble model – scores *s*_*ij*_ and predictions *p*_*ij*_ where *i* represents model number and *j* transcript. Scores can take any number between 0 and 1, while predictions are binary and indicate if the transcript was or was not predicted as a lncRNA. A score greater than or equal to 0.5 would indicate the transcript is predicted as a lncRNA and would have a prediction value of 1. Ensemble models were constructed for random forest and gradient boosting models separately in order to avoid potential correlation of predictions. The four ensemble approaches included both algebraic combiners and voting methods as non-trainable methods, and a stacking generalizer as a meta-learner.

**Fig. 1:**
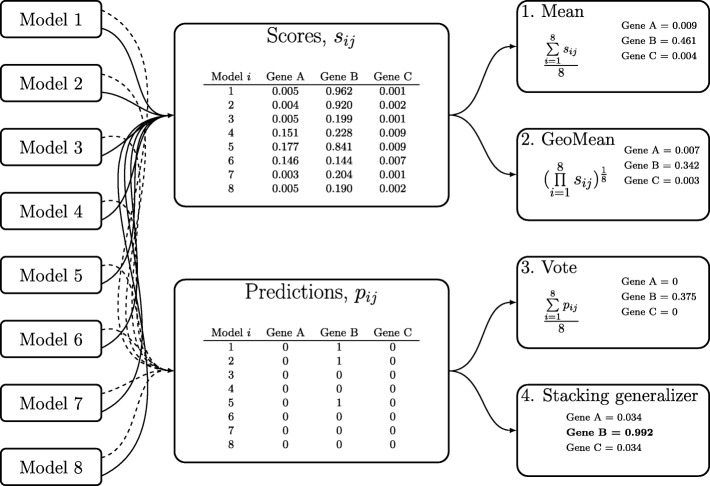
Illustration of ensemble methods. An illustrative example of all four ensemble methods: arithmetic mean, geometric mean, majority vote and the stacking generalizer. Real examples from three different genes are given: gene A represents AT5G44470 a predicted protein, gene B represents At43G09922.1 *IPS1* a known lncRNA, and gene C represents At2G18130.1 a known protein coding gene, *AtPAP11*. Note the final stacking generalizer score of gene B compared to the individual model scores for the gene

The four ensemble methods are described as follows and are illustrated in Fig. 1:

1. **Arithmetic Mean**
  1 $$ \frac{1}{n}\sum\limits_{i=1}^{n}s_{ij}  $$
  Where *n*=8, the number of individual models combined into the ensemble approach. The ensemble decision is made from taking the arithmetic mean of each score *s*_*ij*_ from models 1-8 for each gene *j*. The arithmetic mean of scores will act as a new ensemble score, and prediction will be made as described previously.
2. **Geometric mean**
  2 $$ \left(\prod\limits_{i=1}^{n}s_{ij}\right)^{\frac{1}{n}}  $$
  Where *n*=8, the number of individual models combined into the ensemble approach. The ensemble decision is made from taking the geometric mean for each score *s*_*ij*_ from models 1-8 for each gene *j*. The geometric mean of scores will act as a new ensemble score, and prediction will be made as described previously.
3. **Majority vote**
  3 $$ \frac{1}{n}\sum\limits_{i=1}^{n}p_{ij}  $$
  Where *n*=8, the number of individual models combined into the ensemble approach. The ensemble decision depends only on final predictions and is decided on which label (0 or 1) receives the largest vote. The final prediction is made depending on the value of the majority vote score.
4. **Logistic regression**
  This meta learner is trained on a training dataset of 3000 known *Z. mays* protein coding sequences as negative data and the 10-fold cross validation prediction outputs of known lncRNAs as positive data.

Voting, arithmetic mean, and geometric mean ensemble models were evaluated by directly comparing scores of predictions to the known outcomes of validated lncRNAs and 3000 *Z. mays* protein coding sequences. The logistic regression stacking generalizer was evaluated by 10-fold cross validation. Accuracy, sensitivity, specificity, Matthews correlation coefficient (MCC), and AUC values were calculated using a custom R script and the R package caret [34].

### Comparison of predicted lncRNAs to GreeNC and annotation exploration

Transcript sequences of *O. sativa* and *Eutrema salsugineum* were downloaded from Phytozome v10.3 and *A. thaliana* from TAIR10 for direct comparison to GreeNC. LncRNAs predictions by GreeNC of *A. thaliana*, *O. sativa* and *E. salsugineum* were downloaded on June 19, 2017. Annotations from each species were downloaded from Phytozome v12, with extra *A. thaliana* annotation downloaded from Araport v11.

## Results

### Individual random forest and stochastic gradient boosting model construction

#### Feature selection

Researchers have proposed that specific characters in transcript sequences can be useful in lncRNA classification. For example, lncRNAs can be translated into short peptides [11–13], however most validated lncRNAs remain functional in their RNA form with little protein coding potential. The potential for a transcript to be translated into a protein can be predicted by codon bias, often measured by Fickett score, and hexamer usage bias [21]. Mammalian lncRNAs are known to have a lower GC content than protein-coding RNAs [35], and this feature has been used as a defining feature for *A. thaliana* lncRNA prediction in the past [36]. Transposable elements (TEs) are also known to be sources for plant lncRNAs [3]. Based on these studies, 11 features were originally chosen for use in lncRNA classification: mRNA length, ORF length, GC%, Fickett score, hexamer score, alignment identity in SwissProt database, length of alignment in SwissProt database, proportion of alignment length and mRNA length (alignment length:mRNA length), proportion of alignment length and ORF length (alignment length:ORF), presence of transposable element, and sequence percent divergence from transposable element. Using recursive feature elimination as described in the “Methods” section, features that related to transposable elements were removed since inclusion of these features in classifiers decreased prediction accuracy and thus were deemed uninformative for this training data. After feature elimination, nine features were chosen for implementation in individual random forest and gradient boosting models: mRNA length, ORF length, GC%, Fickett score, hexamer score, alignment identity, length of alignment, alignment length:mRNA length, and alignment length:ORF.

#### Individual model configuration and model evaluation

Gradient boosting and random forest models were constructed using eight different negative training datasets for a total of sixteen models (Table 1). Empirically validated lncRNA transcripts were downloaded from databases as described in “Methods” section. To ensure optimal performance of each gradient boosting classifier, proper calibration of multiple hyper-parameters is required. As such, hyper-parameter tuning (learning_rate, max_depth, subsample, and n_estimators) for each gradient boosting model was completed by grid search and 30 iterations of 4-fold nested cross validation with results summarized in Table 2. All random forest models were constructed with the same hyper-parameters; all options were left as default other than n_estimators=5000 and min_samples_leaf = 20.

**Table 2:** Gradient boosting hyper-parameters chosen by grid search for each model

GB Model #	Learning rate	Maxdepth	Subsample	n estimators
1	0.04	10	0.6	100
2	0.04	10	0.6	100
3	0.04	10	0.6	100
4	0.02	8	0.6	100
5	0.02	10	0.6	100
6	0.02	10	0.6	100
7	0.04	10	0.6	100
8	0.04	10	0.6	100

Hyper-parameters were chosen by grid search using 30 iterations of 4-fold nested cross validation. The given hyper-parameters corresponded to models with the highest accuracy values of all given hyper-parameter combinations

After training calibrated models, gradient boosting and random forest models were evaluated individually by 10-fold cross validation by accuracy, specificity, sensitivity and AUC measures for model validation (Table 1). All models performed at or above accuracy, specificity and AUC measures of 0.94, however, sensitivity values ranged from 0.40 to 0.725 (Table 1). Because of this wide range of sensitivity values, four alternative ensemble approaches using combined random forest and gradient boosting models were explored.

### Ensemble classifier construction

To take advantage of the predictive strengths of each random forest and gradient boosting model, ensemble learners for all random forest and all gradient boosting models were constructed. As ensemble classifiers function by combining “diverse” learners [37], only models constructed from different training sets were used in each ensemble classifier to maintain diversity in predictors. In other words, ensemble classifiers were constructed from all eight random forest models and a separate set of ensemble classifiers were constructed from all eight gradient boosting models.

Four types of ensemble classifiers were constructed: a majority vote model, arithmetic means of scores model, geometric means of scores model, and a stacking ensemble model constructed from a logistic regression of model outputs (Fig. 1 and “Methods” section for details).

A final training set comprised of 3000 known *Z. mays* protein coding genes and validated lncRNAs was created. This *Z. mays* training data set was used for training the logistic regression classifier because random forest and gradient boosting models were trained on different data sets (see “Methods” section). For consistency, all four ensemble methods were also evaluated using these data. The arithmetic mean, geometric mean, and majority vote methods were evaluated by comparing ensemble method outputs to true labels, and 10-fold cross validation scores were used to evaluate the logistic regression stacking model. Accuracy, specificity, and AUC values were similar for all ensemble approaches; therefore, the best performing ensemble method was largely determined by both sensitivity and MCC measures (Table 3). Using these values as methods of evaluation, the stacking model constructed from gradient boosting model outputs was found to be the best performing model and was used for the remainder of the study.

**Table 3:** Evaluation measures of random forest (RF) and gradient boosting (GB) ensemble models

ML model type	Ensemble type	AUC	MCC	Accuracy	Sensitivity	Specificity
RF						
	Vote	0.834	0.725	0.944	0.594	0.995
	Arithmetic mean	0.963	0.661	0.941	0.562	0.996
	Geometric mean	0.963	0.706	0.941	0.555	0.997
	Logistic regression	0.835	0.765	0.952	0.665	0.994
GB						
	Vote	0.887	0.797	0.958	0.702	0.995
	Arithmetic mean	0.945	0.786	0.956	0.681	0.996
	Geometric mean	0.940	0.750	0.949	0.601	0.999
	Logistic regression	0.883	0.822	0.963	0.745	0.994

Statistics for vote, arithmetic mean, and geometric mean models were calculated using outputs of models compared to true labels. Logistic regression evaluation statistics were calculated using the scores found by 10-fold cross validation of *O. sativa* training data and validated lncRNA sequences

### Comparison of meta-learner to GreeNC predictions

To assess the overlap of predictions to another plant lncRNA resource, the lncRNAs predicted by the stacking generalizer were compared to an established lncRNA database, GreeNC [38]. This database uses a transcript filtering method, rather than a machine learning approach, where transcripts must meet the criteria of a classic lncRNA in order to be identified as putative lncRNAs. To be considered a lncRNA in the GreeNC database, the transcript must: be larger than 200nt, have an ORF smaller than 120aa, not have a hit in the SwissProt database or be considered non-coding by the Coding Potential Calculator [39], and not be already classified as another class of functional RNA as identified by Rfam.

**Fig. 2:**
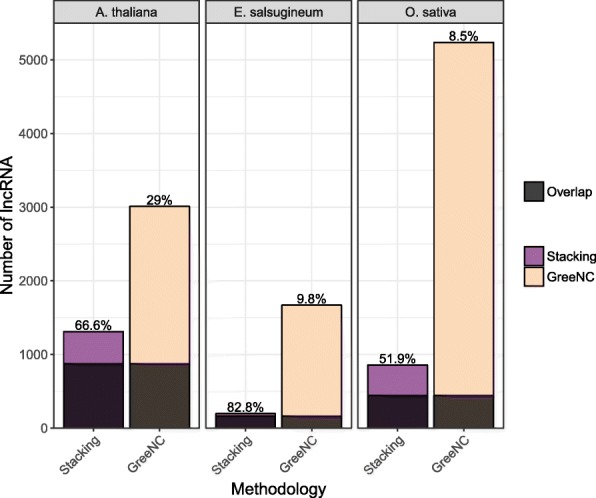
Counts of predicted lncRNAs in *A. thaliana*, *E. salsugineum* and *O. sativa* from the gradient boosting stacking generalizer method and GreeNC database. Counts of predicted lncRNAs in this work from all three species were also compared to predictions recorded in GreeNC. Overlapping predictions of the two methods are represented as shaded bars. The percentages above each bar represent the percent of the total predictions by each method that are shared

Transcript sequences of *O. sativa*, and *E. salsugineum* were downloaded from Phytozome v10.3 and *A. thaliana* sequences from TAIR10 to enable direct comparison to the GreeNC protocol. In total, 1310, 856 and 198 lncRNAs were predicted from *A. thaliana*, *O. sativa*, and *E. salsugineum* respectively, of which 872 (66.6%), 444 (51.9%), and 164 (82.8%) have been previously predicted by GreeNC (Fig. 2). Comparing number of predicted lncRNAs using this method to GreeNC, 1700, 4381, and 1471 fewer lncRNAs are identified in *A. thaliana*, *O. sativa* and *E. salsugineum* using the stacking method. Another 438, 412 and 34 putative lncRNAs were identified using the stacking learner that have not been predicted by GreeNC in *A. thaliana*, *O. sativa*, and *E. salsugineum*.

#### Current annotation of top ranking lncRNAs in *A. thaliana*, *E. salsugineum*, and *O. sativa*

Using the prediction scoring system of this stacking method, the current annotation of the highest ranking lncRNAs from each species was explored. Due to the nature of a logistic regression-type ensemble method, transcripts with similar features will have identical prediction scores. As such, multiple prediction score ties exist in the top ranking transcripts of each species (See Additional file 3 for distribution of lncRNA scores). Using a cutoff of the top three unique prediction scores, annotations of 256, 17 and 94 transcripts in *A. thaliana*, *E. salsugineum*, and *O. sativa* were identified as “top scoring” due to these multiple ties. The majority of predicted lncRNAs in *A. thaliana* were annotated by TAIR as potential natural antisense lncRNAs, pseudogenes, and transposable element related genes (Table 4). Only one transcript from *E. salsugineum*’s top predictions, and two transcripts from *O. sativa*’s top predictions have annotation in Phytozome v12.

**Table 4:** Number of transcripts in annotation categories of top ranking lncRNAs in the *A. thaliana* transcriptome

Annotation category	Number of annotations
Natural antisense lncRNA	64
Pseudogene	75
Transposable element gene	10
Transposase	46
miRNA primary transcript	4
Hypothetical protein	5
Protein	8
Other	8

#### Novel lncRNAs identified by the stacking generalizer

Annotation of the predicted lncRNAs not previously identified by GreeNC from all three species were explored. While all of the newly predicted lncRNAs from *E. salsugineum* and *O. sativa* were annotated as homologs of *A. thaliana* genes, 10 of 34 novel lncRNAs from *E. salsugineum* and 11 of 412 novel lncRNAs from *O. sativa* were annotated specifically as proteins. Of the newly predicted lncRNAs from *A. thaliana*, 417 remain unannotated, with only seven predicted as potential proteins.

## Discussion

Our approach to lncRNA prediction by stacking with logistic regression allows researchers to combine the strengths of various machine learning models without restricting predictions to arbitrary feature cutoffs of a classic lncRNA definition. The flexible nature of this lncRNA prediction tool allows the model to be updated when additional lncRNAs are validated, helping researchers focus on empirical validation of plant lncRNA transcripts. As lncRNA research has previously primarily focused on animal systems with a large emphasis on humans and mice, this tools’ training sets may have a human and mouse bias that is present out of necessity. When more plant lncRNAs are added to the tool’s training set, the human and mouse lncRNA bias that may be found in the model will be reduced. Acting as positive feedback, as more plant lncRNAs are added to the model, the predictions themselves will improve.

To help researchers choose the best lncRNAs for validation, the predictions are ranked. While softwares that rank lncRNA predictions, such as COME [19], do exist, they are trained on a majority of non-empirically validated transcripts adding a potential bias towards non functional transcripts. A combination of ranked predictions and models trained only on true lncRNAs will help ensure researchers focus on the most likely functional lncRNAs

A lower number of identified lncRNAs in comparison to other prediction methods, such as GreeNC, was expected. Using a machine learning classification method, lncRNA predictions were not constrained to arbitrary criteria for this RNA classification. Instead, the classifiers were trained on validated lncRNAs and are expected to identify only true functional lncRNA transcripts. In other words, although transcripts were subjected to less rules for lncRNA identification, the stacking method is expected to have higher accuracy. Further, this work was tested only on sequence information available from Phytozome v10.3 in order to compare predictions directly to GreeNC. Additional transcript sequences available in public repositories, or from researchers’ own sequencing libraries, would add to the number of putative lncRNAs and could be used to improve accuracy. Moreover, COOLAIR and COLDAIR, known *A. thaliana* lncRNAs, are not predicted by GreeNC because the database relies on transcript sequences provided by Phytozome and these transcript sequences were not available in the database at the time of prediction. Our stacking generalizer method for lncRNA prediction is not restricted to a single data source, and allows researchers to calculate a lncRNA score from any transcript sequence, not solely those available from an online repository.

While we expect a lower number of putative lncRNAs than other protocols, of interest is the lower proportion of predicted lncRNAs *E. salsugineum* genome compared to *O. sativa* or *A. thaliana*. A reason for the low lncRNA discovery rate in *E. salsugineum*, could potentially be that plants were not subjected to conditions sufficient for observable lncRNA expression. For example, *IPS1* [8] and *COLDAIR* [9], two well studied *A. thaliana* lncRNAs, are induced by phosphate or cold-related stresses respectively. This hypothesis is supported by Derrien et al. [16] who found human lncRNA expression to be at low levels in a condition, tissue and developmental state specific manner. It is also possible that there exists natural variation in the numbers of putative lncRNAs in different species. Further investigation on the number of putative lncRNA and their relationships to plant growth conditions for transcriptome sequencing of multiple plant species is currently underway.

Although the quantity of detected lncRNAs was low in *E. salsugineum*, the quality of putative lncRNAs in all three species is high, demonstrating that this tool can accurately classify transcripts no matter size or quality of input transcript sequence data. When exploring the annotations of the top scoring predictions in *A. thaliana*, the majority of transcripts were annotated as potential natural antisense lncRNA, pseudogenes, transposable elements, small RNA primary transcripts, or remain computationally predicted as hypothetical proteins (Table 4). Pseudogenes remain poorly understood, however there is evidence of pseudogene derived lncRNAs regulating their parental genes [40], making pseudogene derived lncRNAs targets of potential regulatory interest. Transposable elements are another known source of lncRNAs, particularly in vertebrates [41] and long intergenic non-protein coding RNAs in plants [3]. This study did not find evidence that features related to transposable elements were helpful at predicting plant lncRNAs as the addition of transposable related features decreased the quality of lncRNA predictions. However, exploration of the training data used for model creation indicates that only 19 of the 436 (4.4%) validated lncRNAs show evidence of transposable element association. Of this minor group of transposable element associated lncRNAs, none were from plant species. Nonetheless, the tool did not favour lncRNAs that are not associated with transposable elements, as the tool remained successful at identifying these types of transcripts. Additionally, as novel lncRNAs are validated and added to this tool, an update to the models’ feature selection step may be required, and may lead to future inclusion of transposable element associated characters. However, by not including transposable element information, the computational time for data preprocessing before transcript classification is significantly reduced to minutes from days as RepeatMasker is no longer needed.

Features of secondary RNA structure have previously been used in other RNA classifiers, such as nRC [42] and GraPPLE [43], that are used to classify RNAs into functional categories. These classifications include RNAs such as miRNAs, tRNAs, rRNA, ribozymes, and riboswitch domains, all of which have conserved secondary structures. Rather than using sequence homology, commonly used with protein coding genes, structural homology has previously been used in lncRNA functional prediction, and identification [5]. However, a lack of secondary structure conservation in animal lncRNAs with conserved sequences (*e. g. HOTAIR, ncSRA and Xist*) was recently observed [44]. As structural conservation may not be as pervasive in lncRNA classification as previously thought, we did not include structural features in our ensemble learner. A lack of structural features allows the predictor to identify a wide variety of lncRNAs and does not limit the predictor to the structures of the small number of validated plant lncRNAs available. An additional test was completed to ensure our predictor, lacking structural features, did not merely distinguish non-coding transcripts from protein coding genes. By comparing the results of the ensemble learner to predicted CPAT protein coding probabilities [21], our ensemble method was able distinguish between other CPAT-predicted non-coding transcripts and likely lncRNAs (Additional file 4: Table S2). A portion of putative lncRNAs in all three plant species are also predicted to be protein coding and may encode small regulatory peptides.

High quality lncRNA predictions from this method require sequences from fully processed transcripts and cannot be predicted directly from genomic sequences. Nevertheless, potential lncRNA sequences of interest are typically more accessible by transcriptome sequencing rather than complete genome sequencing, which remains technically challenging for crop plants with large and/or polyploid genomes. This tool is flexible and can be used to identify lncRNAs from all transcriptional units of an organism, or to check the lncRNA score of a single transcript. Furthermore, as mentioned in their summary, Kang et al. [22] suggest that researchers should now consider working on uncovering the biological implications of lncRNAs rather than solely using computational tools for transcript classification. We agree that future work should centre around using software to also further knowledge on these types of transcripts. Due to the diversity of these transcripts, there is increasing need for classification of lncRNAs into categories based on mechanism and function, as well as continuation of empirical validation, particularly for plants. Once validated, not only can novel lncRNAs mechanisms be explored, but their features can be added to this tool for further improvement in lncRNA prediction.

## Conclusion

For this machine learning based tool for lncRNA prediction, we have used only empirically validated lncRNAs for training. Although lncRNAs from multiple species were used, our tool identified putative plant lncRNAs with high scores. Ranking of lncRNA predictions should improve the confidence by which gene products meriting validation are selected for empirical testing. The machine learning structure and its open source availability allows for the flexible inclusion of validated lncRNAs as our knowledge of this class of RNA improves. An important consideration of this tool is that it is not constrained by preconceived rules that may or may not appropriately classify lncRNA properties. As Kung et al. [1] suggest, setting rules for the detection of these non-conforming transcripts could be detrimental due to the diversity in functionality, structure, expression and mechanism of these transcripts. Accordingly, our stacking generalizer model based on gradient boosting models will facilitate lncRNA identification without imposing arbitrary rules for lncRNA detection.

## Additional files

Additional file 1 Non-coding RNA search terms. Terms used to search for organism specific non-coding sequences on RNA central. (ZIP 16500 kb)

Additional file 2 Random protein training data sets, lncRNA data sets. Fasta files of protein coding and lncRNA sequences in data sets used for training machine learning classifiers. (TXT 0.07725 kb)

Additional file 3 Distribution of predicted lncRNA scores. Figure and table of distribution of scores. (PDF 58 kb)

Additional file 4 Comparison of predicted lncRNAs to CPAT results. Table of results and explanation of additional test. (PDF 31 kb)

## Abbreviations

AUC: Area under the curve
lncRNA: Long non-protein coding RNA
MCC: Matthews correlation coefficient
ORF: Open reading frame
TE: Transposable element
