# Supplementary material for: Prediction of plant lncRNA by ensemble machine learning classifiers
Source: BMC Genomics. 2018 May 2;19:316. doi: 10.1186/s12864-018-4665-2 (PMC5930664; doi:10.1186/s12864-018-4665-2)
Supplement: Supplementary file 3 — Distribution of predicted lncRNA scores. Figure and table of distribution of scores. (PDF 58 kb) [file 12864_2018_4665_MOESM3_ESM.pdf]

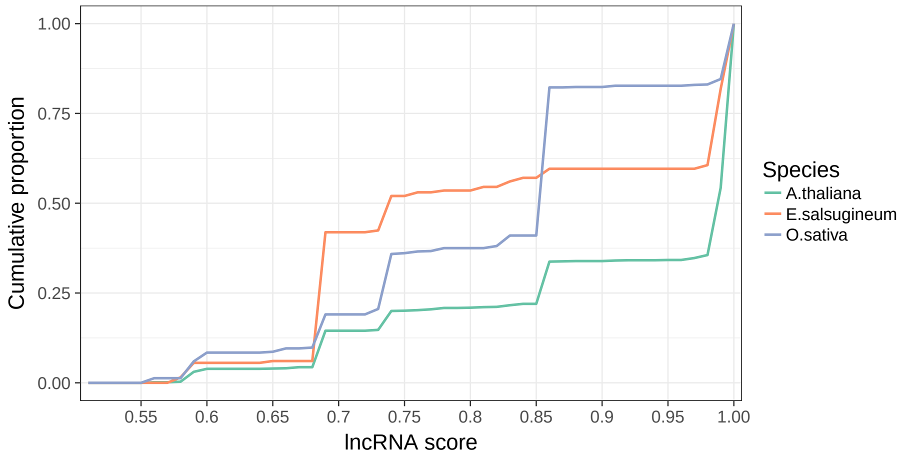

Supplemental Figure 1: **Cumulative proportions of lncRNA scores in *A. thaliana*, *E. salsuginiem*, and *O. sativa*. found using the gradient boosting stacking generalizer**. The figure depicts the proportions of lncRNAs that are predicted as a lncRNA equal to or less than a particular score.

Supplemental Table 1: The distribution of lncRNA prediction scores in *A. thaliana*, *E. salsugineum*, and *O. sativa*

Score	*A. thaliana*	*E. salsugineum*	*O. sativa*
0.50-0.55	0	0	0
0.55-0.60	51	11	72
0.60-0.65	1	1	2
0.65-0.70	138	71	89
0.70-0.75	73	20	146
0.75-0.80	11	3	12
0.80-0.85	14	7	30
0.85-0.90	156	5	354
0.90+	866	80	151
Total	1310	80	148
