# Supplementary material for: Prediction of plant lncRNA by ensemble machine learning classifiers
Source: BMC Genomics. 2018 May 2;19:316. doi: 10.1186/s12864-018-4665-2 (PMC5930664; doi:10.1186/s12864-018-4665-2)
Supplement: Supplementary file 4 — Comparison of predicted lncRNAs to CPAT results. Table of results and explanation of additional test. (PDF 31 kb) [file 12864_2018_4665_MOESM4_ESM.pdf]

Supplemental Table 2: Ensemble predictor has no preference for coding to noncoding sequences.

	CPAT prediction:			
	Coding		Noncoding	
CREMA prediction	lncRNA positive	lncRNA negative	lncRNA positive	lncRNA negative
Species				
A. thaliana	243	37279	1067	2314
O. sativa	133	49325	723	1762
E. salsugineum	44	28079	154	1161

Transcripts were identified as either protein coding or non-coding using the CPAT software. Coding probability cutoffs were calculated by intersect of sensitivity and specificity via 10-fold cross validation. A coding probability cut off of 0.38 was used for *A. thaliana* and 0.52 for both *O. sativa* and *E. salsugineum*.
